# Red blood cell transfusion-related dynamics of extracellular vesicles in intensive care patients: a prospective subanalysis

**DOI:** 10.1038/s41598-023-48251-w

**Published:** 2024-01-09

**Authors:** Pierre Raeven, Katharina Karlhofer, Larissa S. Sztulman, Jonas Brugger, Konrad Hoetzenecker, Christoph Domenig, Gerda Leitner, Martin Posch, David M. Baron, Andreas Spittler

**Affiliations:** 1 Division of General Anesthesia and Intensive Care, Department of Anesthesia, General Intensive Care, and Pain Management, Medical University of Vienna, Vienna, Austria; 2 Division of Visceral Surgery, Department of Surgery, and Core Facility Flow Cytometry, Medical University of Vienna, Vienna, Austria; 3 Division of Visceral Surgery, Department of Surgery, Medical University of Vienna, Vienna, Austria; 4 Center for Medical Statistics, Informatics, and Intelligent Systems, Section for Medical Statistics, Medical University of Vienna, Vienna, Austria; 5 Department of Thoracic Surgery, Medical University of Vienna, Vienna, Austria; 6 Division of Vascular Surgery, Department of Surgery, Medical University of Vienna, Vienna, Austria; 7 Department of Blood Group Serology and Transfusion Medicine, Medical University of Vienna, Vienna, Austria

**Keywords:** Translational research, Flow cytometry

## Abstract

Extracellular vesicles (EVs) accumulate during packed red blood cell (PRBC) storage. To date, the involvement of EVs in transfusion-related immunomodulation (TRIM) has not been prospectively evaluated in intensive care unit (ICU) patients. This was a prospective subanalysis of a recent observational feasibility study in postoperative ICU patients after: (1) open aortic surgery (Aorta), (2) bilateral lung transplantation (LuTx), and (3) other types of surgery (Comparison). Patient plasma was collected three times each before and after leukoreduced PRBC transfusion at 30-min intervals. The total number of EVs and EVs derived from erythrocytes (EryEVs), total platelets (total PEVs), activated platelets, granulocytes (GEVs), monocytes, and myeloid cells in PRBC samples and patient plasma were analyzed by flow cytometry. Statistical analysis was performed by Spearman’s correlation test, linear mixed models and pairwise comparisons by Wilcoxon matched-pairs test. Twenty-three patients (Aorta n = 5, LuTx n = 9, Comparison n = 9) were included in the final analysis. All EV subgroups analyzed were detectable in all PRBCs samples (n = 23), but concentrations did not correlate with storage time. Moreover, all EVs analyzed were detectable in all plasma samples (n = 138), and EV counts were consistent before transfusion. Concentrations of total EVs, EryEVs, total PEVs, and GEVs increased after transfusion compared with baseline in the entire cohort but not in specific study groups. Furthermore, the change in plasma EV counts (total EVs and EryEVs) after transfusion correlated with PRBC storage time in the entire cohort. Extracellular vesicles were detectable in all PRBC and plasma samples. Individual EV subtypes increased after transfusion in the entire cohort, and in part correlated with storage duration. Future clinical studies to investigate the role of EVs in TRIM are warranted and should anticipate a larger sample size.

**Trial registration:** Clinicaltrials.gov: NCT03782623.

## Introduction

As part of the “storage lesion”, packed red blood cells (PRBCs) secrete bioactive inflammatory mediators during storage and processing^1–3^. After transfusion, these mediators can influence the immune system, a process termed transfusion-related immune modulation (TRIM)^4–7^. The mechanisms of TRIM are not fully understood^8–10^, but extracellular vesicles (EVs) accumulating in PRBCs might play a role^11,12^. Immunomodulation might particularly affect vulnerable patients, such as those admitted to the ICU after undergoing major surgery. These patients make up a significant proportion of PRBC recipients and may be in an activated, suppressed, or mixed immunological state^13,14^.

Extracellular vesicles are a heterogeneous group of subcellular particles lacking a functional nucleus, and can be distinguished based on size, surface markers, content, development from parental cells, and biological function^15^. The most recent definition of EVs is based on the updated International Society of Extracellular Vesicles guidelines published in 2018^16^. A detailed characterization of EV concentrations in PRBCs and changes of EV concentrations in patient plasma after transfusion could support future personalized transfusion strategies in the ICU^17^. Therefore, future research should focus on understanding the mechanisms of TRIM by analyzing the effects of specific blood products on individual patients with potential risk factors^5^.

Concentrations of EVs in plasma before and after transfusion in combination with EV concentrations in PRBCs have been studied in healthy volunteers^12^. To the best of our knowledge, one clinical report has evaluated EVs in plasma before and after allogeneic transfusion in retrospectively selected ICU patients^18^. However, EV concentrations in PRBCs were not analyzed in this study. This may be due to the arduous prospective recruitment of critically ill patients receiving PRBC transfusion, as reported recently^19^.

We have recently conducted a feasibility study investigating the effect of PRBC transfusion on dynamics of eicosanoids in ICU patients^20^. The aim of this subanalysis was to provide prospective exploratory data for future studies on transfusion-related dynamics of EVs in a postoperative ICU setting. We hypothesized that (1) EVs are detectable in PRBCs and their number correlates with storage time; (2) EVs are detectable in plasma of ICU patients with stable concentrations before PRBC transfusion and an increase after PRBC transfusion; (3) the increase of EV concentrations in plasma after PRBC transfusion correlates with EV concentrations in transfused PRBCs.

## Methods

### Ethics approval and accordance statements

All experimental protocols were approved by the Institutional Review Board of the Medical University of Vienna (Nr. 1595/2018). All methods were carried out in accordance with relevant guidelines and regulations. The manuscript was written using the STrengthening the Reporting of OBservational studies in Epidemiology and CONSORT checklists^21^.

### Informed consent statement

Informed consent was obtained from all participants.

### Human transplantation statement

Transplanted lungs were procured via the Lung Transplantation Program at our institution. No organs/tissues were procured from prisoners.

### Study design and sample size estimation

All transfused PRBC units in this study were leukoreduced. This was a predefined subanalysis of our recent feasibility study examining transfusion-related eicosanoid abundances in ICU patients^20^. Briefly, we recruited postoperative intensive care patients, including patients taking acetylsalicylic acid (ASA) or immunosuppressants, to study the effects of these drugs. Consequently, three groups were defined: (1) patients treated with ASA after aortic surgery (Aortic group); (2) patients admitted after bilateral lung transplantation with immunosuppressants (i.e., prednisolone and tacrolimus) but without ASA treatment (LuTx group); (3) patients not treated with either ASA or immunosuppressants and admitted after other types of surgery (Comparison group). Following a recent study in healthy volunteers using n = 6 per group^12^, we planned to eventually include 10 patients per group. Because we anticipated that not every patient screened would require PRBC transfusion, we anticipated a 2-year screening/recruitment period based on transfusion rates at our hospital, from December 1, 2018 to November 30, 2020.

### Inclusion and exclusion criteria

We included 18- to 99-year-old patients who received PRBC transfusion after postoperative admission to the ICU. In the original study, the following drugs were considered to have confounding effects on plasma eicosanoid dynamics^22,23^ and were therefore excluded for recruitment: celecoxib, etoricoxib, parecoxib, ibuprofen, diclofenac, naproxen, and cysteinyl leukotriene receptor antagonists (e.g., montelukast) in all groups, acetylsalicylic acid and protamine for heparin reversal in the LuTx and Comparison groups, and glucocorticoids given within 24 h of transfusion in the Aortic and Comparison groups. Other exclusion criteria were pregnancy and a period of less than 12 h since the last PRBC transfusion. Because we did not expect ASA to have an effect on EVs in plasma, the drug was not excluded from recruitment.

### Sample collection

**Figure 1:**
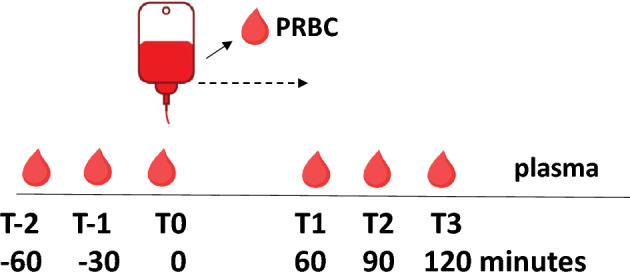
Overview of sampling time points. Prior to transfusion of one unit of stored packed red blood cells (PRBC) over 1 h ± 5 min, plasma was sampled at 60, 30 and 0 min before transfusion (T-2, T-1 and T0, respectively). At 60, 90 and 120 min after the start of the PRBC transfusion (T1, T2 and T3, respectively), plasma was again sampled. Additionally, one sample was drawn directly out of the PRBC unit. The transfusion period is indicated by the dashed line. Modified, previously published Figure.

Figure 1 shows the sampling procedure. Using 3.5 ml of 3.2% sodium citrate anticoagulant, one sample was collected directly from the transfusion unit, while peripheral blood was collected via indwelling arterial catheters. Blood was collected at 60 min, 30 min, and immediately before transfusion (T-2, T-1, and T0, respectively) to test for baseline EV variations. Then, one unit (250 ml) of stored PRBCs was transfused over 1 h ± 5 min. Subsequently, blood samples were collected at 60, 90, and 120 min after the start of PRBC transfusion (T1, T2, and T3, respectively). Platelet-free plasma (PFP) was obtained from PRBCs and patient blood samples by two consecutive centrifugation steps at room temperature: first at 2500×*g* for 15 min and then the supernatant was centrifuged again at 13,000×*g* for 5 min.^24^ The PFP was divided into 200 µl aliquots, snap frozen with liquid nitrogen and stored at − 80 °C until further analysis.

### Flow cytometry analysis

EVs were analyzed according to our previously published protocol^24^ with modifications. PFP was thawed in a water bath at 37 °C and immediately processed for immunostaining. For this purpose, 10 µl of PFP were treated with monoclonal antibodies directed against cluster of differentiation (CD)235a-R-phycoerythrin (PE, clone 11E4B-7-6, Beckman Coulter, Brea, CA), CD62p (PE, clone REA389, Milteny, Bergisch Gladbach, Germany), CD15 (PC7, clone W6D3, Biolegend, San Diego, CA), CD41a (PC7, clone P2, Beckman Coulter, Brea, CA), CD66b (PE, clone REA306, Milteny, Bergisch Gladbach, Germany), CD14 (PC7, clone HCD14, Biolegend, San Diego, CA). Then, 10 µl of Lactadherin (LA; AF647, CellSystems, Troisdorf, Germany) in PBS and 50 µl of the intracellular fluorescent dye calcein AM (Calcein Green, Thermo Fisher Scientific, Waltham, MA) in dimethyl sulfoxide were added and incubated for 30 min in the dark on ice. Subsequently, 50 µl of paraformaldehyde (4%) was added to all samples which were than diluted with PBS to a total volume of 1000 µl. Table S1 shows the compilation of antibodies and fluochromes used in this study. To avoid swarming effects^25^ the samples were initially diluted 1:2 and measured at a maximum of 2500 events per second with further dilution when necessary.

To detect EVs, a Boolean “or” combination of two trigger signals was specified; either LA AF647 or calcein AM (channel FL1) was used, depending on which signal appeared to be above threshold. Total EV number was then defined as the sum of particles that were double positive for LA and calcein AM (i.e., LA^+^calcein AM^+^), single positive for LA (i.e., LA^+^calcein AM^−^), and single positive for calcein AM (i.e., LA^−^calcein AM^+^). Total EVs were further characterized by surface staining for the following subpopulations: EVs from erythrocytes (EryEVs, CD235a^+^), nonactivated platelets (PEVs, CD41^+^CD62p^−^), activated platelets (activated PEVs, CD41^+^CD62p^+^ and CD41^−^CD62p^+^), granulocytes (GEVs, CD66b^+^), monocytes (MEVs, CD14^+^), and myeloid cells (CD15^+^). Prior to staining, the antibody mixture was centrifuged at 20,000×*g* for 30 min to remove antibody aggregates.

Flow cytometry was performed using a CytoFLEX LX flow cytometer (Beckman Coulter). For this study, the flow cytometer had a five-laser path with lasers of 355 nm, 405 nm, 488 nm, 561 nm, and 638 nm. A sample incubated with Triton X served as a lysed autofluorescence control. For daily routine, the CytoFLEX was turned on according to the manufacturer’s recommendations. The instrument was then rinsed with double distilled water from a freshly opened bottle for 30 min. We also used double distilled water as sheath fluid reagent.

### Statistical analysis

Spearman’s rho was determined to evaluate the correlations between EV concentrations in PRBCs and storage time and also between EV concentrations in PRBCs and the change in plasma EV concentrations after PRBC transfusion. EV concentrations were analyzed for normality and expressed as median and interquartile range. Differences between time points compared with baseline values in the entire cohort were tested with the Wilcoxon matched-pairs test. For the pairwise comparison, the 3 baseline values (i.e., T-2, T-1, and T0) of each patient were averaged.

To examine whether EV concentrations in each study group changed over time, linear mixed models were fitted with the concentration of an EV subgroup as the dependent variable and the study group and the interaction of study group and time as numeric variables (0, 1, 1.5, 2 h after transfusion) as fixed parameters. Baseline concentrations were treated as if they were all obtained at time T0 (because we assume that there is no temporal trend before the intervention). Random intercepts and slopes were considered for each patient. We tested whether the slopes were significantly different from 0. No correction for multiple testing was applied. Therefore, all p values are descriptive and hypothesis-generating in nature. Statistical analysis was performed using Prism 9 (GraphPad) and R version 3.6.1 or higher (R Development Core Team, https://www.R-project.org/). Mixed models were computed using the lmerTest package^26^.

## Results

### Patient recruitment

**Figure 2:**
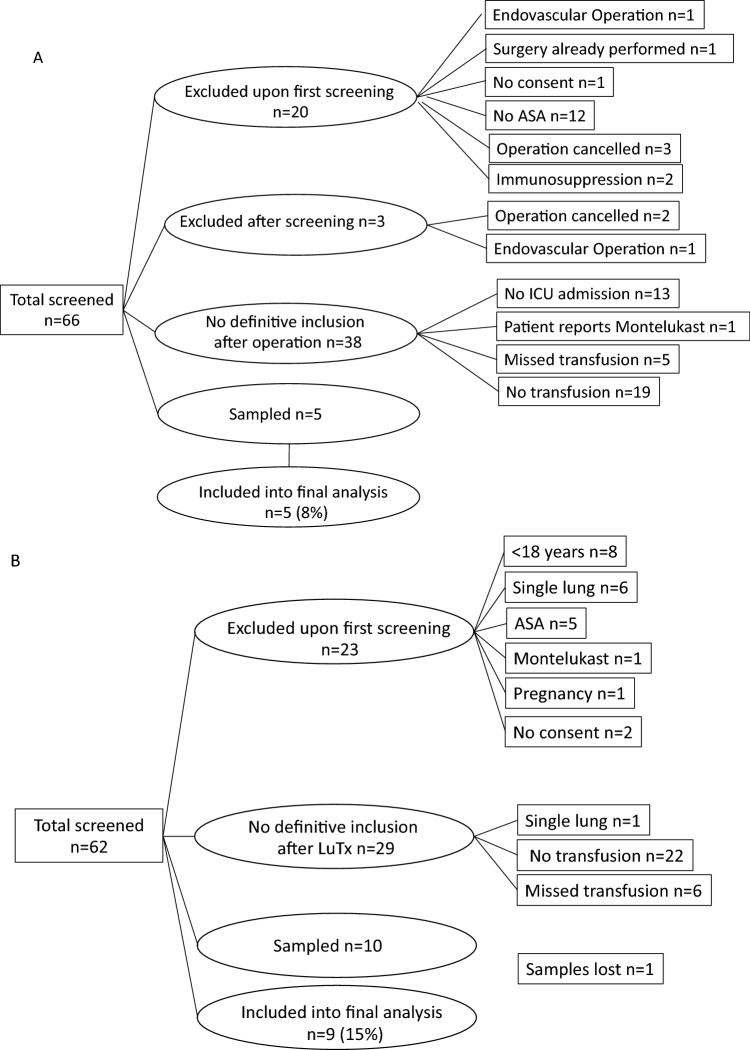
Flowcharts of patient recruitment. Patients scheduled for aortic surgery (**A**) or bilateral lung transplantation (**B**) were pre-screened, and informed consent was obtained before surgery. Definitive inclusion into the study was performed after surgery. *LuTx* bilateral lung transplantation, *ASA* acetyl salicylic acid. Figure has been published previously^20^ and was modified according to patients included in this subanalysis.

Figure 2 shows the flow diagram of patients in the Aorta and LuTx groups. Sampling was performed in 5 patients in the Aorta group and 10 patients in the LuTx group. Simultaneously, ICU patients undergoing general surgery were screened daily, and 9 postoperative ICU patients (Comparison) were recruited and sampled during the same study period. Samples from one patient in the LuTx group (patient No. 10) became contaminated during analysis after recruitment had ended. Therefore, 23 patients (Aorta n = 5, LuTx n = 9, Comparison n = 9) were included in the final analysis.

Because of the COVID-19 pandemic, operative capacity for patients requiring postoperative treatment at the ICU was temporarily limited at our institution. Therefore, recruitment was terminated on March 19, 2020.

### Patient characteristics

Table 1 and Table S2 depict the main characteristics of patients included into the study.

**Table 1:** Main patient characteristics.

Group	Pat ID	Age	Sex	Diagnosis	Operation	POD	PRBC age (days)	PRBCs transfused during surgery (n)	Total PRBCs in the ICU (n)
Aorta	6	78	m	Abdominal aortic aneurysm	Bifemoral prothesis	1	32	0	3
	34	65	m	Peripheral arterial vascular disease	Axillofemoral bypass	1	11	2	2
	36	77	m	Abdominal aortic aneurysm	Bifemoral prothesis	2	24	0	2
	48	82	f	Abdominal aortic aneurysm	Bi-iliacal prothesis	1	17	3	4
	81	81	m	Abdominal aortic aneurysm	Aortic replacement	2	29	1	4
LuTx	11	56	m	Chronic obstructive pulmonary disease	LuTx	2	33	4	3
	12	32	f	Idiopathic pulmonary hypertension	LuTx	1	23	9	6
	14	59	f	Chronic obstructive pulmonary disease	LuTx	3	36	2	1
	15	38	m	Cystic fibrosis, transplant dysfunction	Re-LuTx	3	31	7	2
	25	25	f	Cystic fibrosis	LuTx	3	29	10	1
	45	26	m	Cystic fibrosis, organ rejection	Re-LuTx	14	20	16	10
	49	48	m	Cystic fibrosis, transplant dysfunction	Re-LuTx	8	18	22	5
	64	37	m	Acute respiratory distress syndrome	LuTx	6	23	17	5
	73	33	m	Cystic fibrosis	LuTx	3	20	5	2
Comparison	17	36	m	Abdominal wall seroma	Multiple	5	22	7	6
	20	84	m	Neoplasma vesicae	Cystectomy	3	25	0	1
	43	56	m	Pericardial effusion	Pericardial drainage	4	33	0	17
	46	77	m	Status post subhepatic hematoma	Wound management	1	29	0	6
	61	59	m	Thymoma	Thymectomy	44	18	13	20
	68	59	m	Renal cell carcinoma	Partial nephrectomy	1	17	3	5
	79	72	f	Stomach perforation	Gastrectomy	1	13	1	1
	80	62	m	Neoplasma vesicae	Cystectomy	1	25	0	2
	87	59	m	Peripheral arterial vascular disease	Thrombectomy	2	20	0	3

Table has been published previously^20^ and was modified according to patients included in this subanalysis.

*Pat ID* patient identification number, *POD* postoperative day at time point of transfusion, *PRBC* packed red blood cell, *d* days, *n* count, *ICU* intensive care unit, *LuTx* bilateral lung transplantation, *Re-LuTx* repeated bilateral lung transplantation, *m* male, *f* female.

### EV concentrations in PRBCs and correlation with storage time

TableS3.xlsx shows the concentrations of EVs in PRBC units (and also patient plasma) at all predefined time points. EVs from all subgroups analyzed were ubiquitously detectable in each PRBC sample. Whereas 4% of total EVs were EryEVs, EVs derived from other cell types (especially granulocytes and platelets) accounted for less than 1% of total EVs. Figure S1 shows the EV concentrations in the transfused PRBCs plotted against the storage time of the respective PRBCs. Of all subgroups analyzed, Ery-EVs (rho = 0.28, p = 0.2) and PEVs (rho = 0.24, p = 0.3) showed the strongest tendency towards a correlation between EVs concentrations in the PRBCs and storage duration. However, none of the EVs in the PRBCs supernatant significantly correlated with PRBCs storage duration. All observed correlation coefficients were between − 0.13 and 0.28.

### Variation of EV concentrations in plasma before transfusion

Table 2 shows the range of variation of EV concentrations in the three plasma samples collected before transfusion. EV concentrations were never lower than 0.25-fold and never higher than 1.87-fold of the mean values of these three samples. Within these ranges, EryEVs (CD235^+^), PEVs (CD41^+^), and MEVs (CD14^+^) showed the greatest variation within the 60 min before transfusion.

**Table 2:** Fluctuations of EVs counts per µl at baseline (T-2, T-1 and T0).

Extracellular vesicle subtype	Lower limit of range	Upper limit of range
Total EVs (LA^+^calcein AM^+^ + LA^+^calcein AM^−^ + LA-calcein AM^+^)	0.69-fold	1.34-fold
Erythrocyte-derived (CD235^+^) EVs	0.28-fold	1.87-fold
Platelet-derived (CD41^+^CD62p^−^) EVs	0.25-fold	1.79-fold
Activated platelet-derived (CD41^+^CD62p^+^ and CD41^-^CD62p^+^) EVs	0.53-fold	1.62-fold
Granulocyte-derived (CD66b^+^) EVs	0.62-fold	1.44-fold
Monocyte-derived (CD14^+^) EVs	0.38-fold	1.79-fold
Myeloid cell-derived (CD15^+^) EVs	0.64-fold	1.54-fold

Values depict change of values relative to the non-logarithmic average of all three baseline values.

*LA* lactadherin, *EVs* extracellular vesicles, *CD* cluster of differentiation.

### Change of EV concentrations after PRBC transfusion vs. baseline in the whole cohort

**Figure 3:**
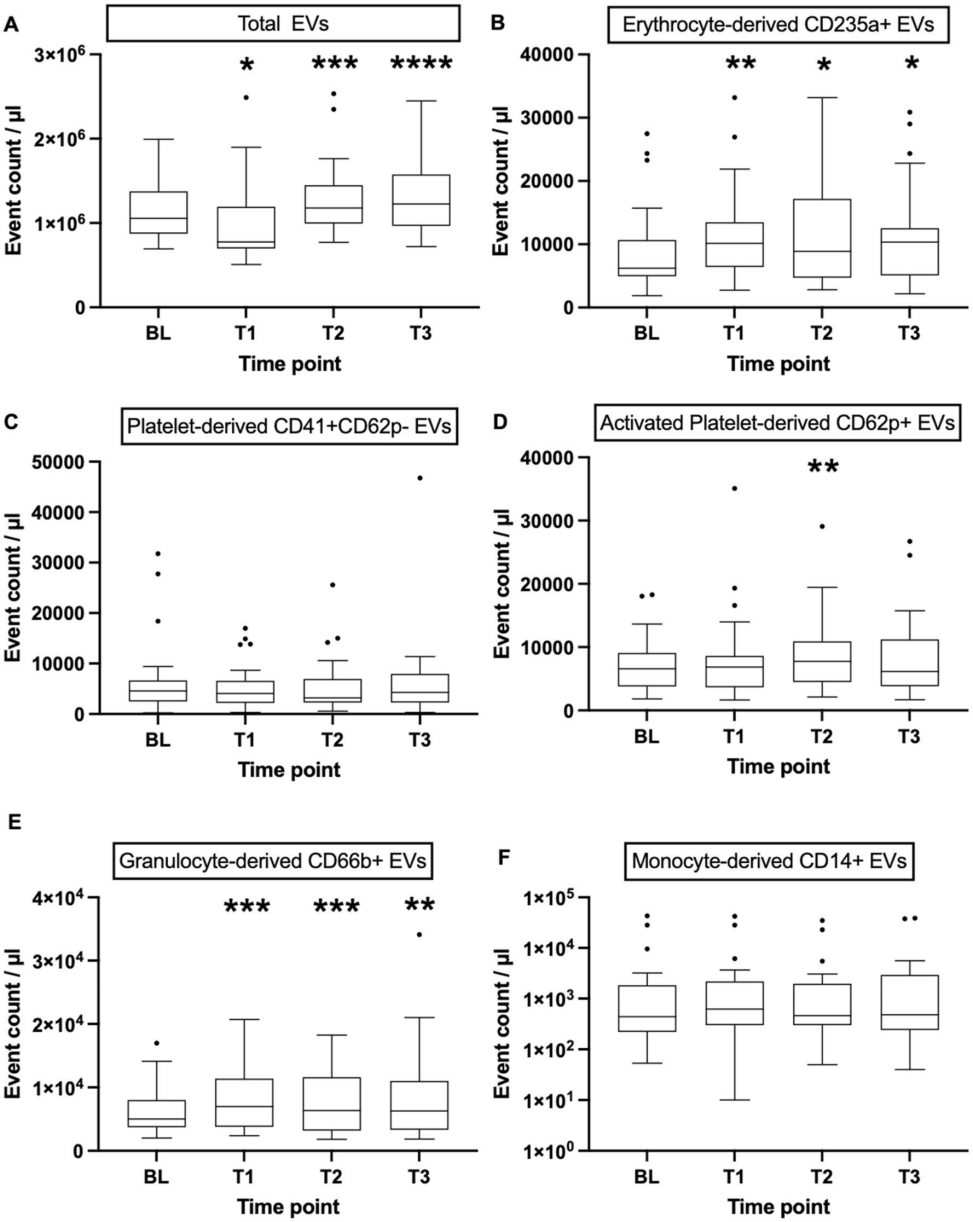
Plasma EV counts in the whole cohort. Before transfusion, plasma samples were drawn at three time points and averaged to one baseline value. After transfusion, plasma was sampled immediately (T1), 30 min (T2), and 60 min (T3) after the end of transfusion. Boxes represent IQR with median. The upper and lower whisker extends to lowest/highest value, respectively within 1.5 times the IQR from the hinges. Black dots show values outside 1.5 times the IQR from the hinges. N = 23 per time point. *p < 0.05, **p < 0.01, ***p < 0.001, ***p < 0.0001 versus BL. *LA* lactadherin, *CD* cluster of differentiation, *EVs* extracellular vesicles, *BL* baseline, *PLT* platelet.

Figure 3 shows the dynamics of EV concentrations in the entire cohort (n = 23). Total EV counts (Fig. 3A) initially decreased at T1, but subsequently increased at time points T2 and T3 compared to baseline. Conversely, EryEV counts (Fig. 3B) and GEV counts (Fig. 3E) increased compared to baseline at all time points after transfusion. For activated PEV counts (Fig. 3D), the increase from baseline reached statistical significance only at T2. For the remaining EV subgroups analyzed, no significant differences in EV levels were observed after PRBC transfusion compared to baseline.

### EV time courses in individual patients analyzed per group

To gain better insight into whether the changes observed in the entire cohort were attributable to specific individual patients or patient groups, we plotted the time courses of EV dynamics for individual patients stratified by study group (Fig. S2). Indeed, the time courses for individual patients showed heterogeneous dynamics. Moreover, an increase in EryEVs after transfusion was observed in the comparison and LuTx groups, whereas the values tended to decrease in the Aortic group. Furthermore, the increase in GEVs and activated PEVs in the entire cohort may have been driven primarily by an increase in the Comparison group.

### Correlation of change of EV plasma levels and levels in stored PRBCs

Table S4 shows the correlation of the change in EV plasma concentration at baseline with post-transfusion levels and with their concentration in transfused PRBCs. Total EV concentrations in PRBC supernatant correlated with the change in plasma levels at T2 as well as T3 versus baseline, respectively (Spearman’s rho ≥ 0.5, p < 0.01). For EryEVs, the change in plasma levels between baseline and T1 also correlated moderately with EryEVs levels in PRBC supernatant.

## Discussion

In this subanalysis of our recent feasibility study^20^, we prospectively investigated the dynamics of EV concentrations before and after transfusion in postoperative ICU patients. Specific EV subtypes detectable in PRBC units increased in the plasma of the transfused patients. Interestingly, the increase in plasma EV concentrations after transfusion correlated with PRBC storage time.

All EV subtypes investigated in our study were ubiquitously detectable in PRBCs and plasma from ICU patients. This technical and analytical robustness enabled us to perform statistical analysis despite a small sample size. However, it should be noted that the statistics are descriptive, hypothesis-generating in nature. Thus, the exploratory design of this study requires cautious interpretation. In contrast to previous studies^11,12^ and our hypothesis, the number of EVs in PRBCs and their storage duration did not correlate in our study. Although this could be related to the small sample size or differences in donor characteristics, another reason could be our particular approach. Previous, preclinical investigations report increased EV concentrations in PRBC units stored for 35 days versus those stored for 2 days using only 6 samples per storage duration group^12^. In our clinical setting, we paired the EV counts in the PRBC units (n = 23) with their respective (non-predefined) storage duration at the time of transfusion for correlation analysis, which may require a larger sample to avoid a type 2 statistical error. Indeed, another study applying a similar approach using 126 PRBC donors (and including fresh PRBC samples) suggests an accumulation of total EVs, EryEVs and total PEVs^11^.

Because plasma levels of mediators can change rapidly in the ICU setting, we also studied the consistency of EV levels before transfusion. The fluctuation of plasma EV counts was within a predefined range of 0.2- to twofold of the mean values of the three baseline samples. To our knowledge, serial measurements of EVs in plasma (from postoperative ICU patients) within a 60-min period have not been reported previously. However, several studies have described baseline plasma EV values at selected time points in healthy volunteers before and after infusion of lipopolysaccharide^12^, in patients before coronary artery bypass graft surgery^27^, and in ICU patients^18^. Unfortunately, the different EV analysis methods used in aforementioned studies preclude direct comparison of baseline values. This is a well-known problem in EV research, even though attempts have been made to harmonize definitions, analyses, and reporting^16,28,29^. Approximately 60% of EVs with a size of ≥ 200 nm are intact and (still) PS negative^30^, and might therefore be missed if only positivity for PS is used for identification. For EV detection, we have therefore moved from using only annexin or LA as a marker to combining LA and calcein AM to allow more accurate identification^24,31^.

Due to this double-identification of total EVs, one would expect more total EV counts in our study as compared to previous investigations. Even though lack of raw data from previous investigations precludes exact comparison, the total number of EVs in our study was indeed clearly higher than that in studies not using calcein AM: approximately tenfold higher when compared to healthy subjects, (only LA as marker)^12^, and > 5000-fold higher than in ICU patients (annexin as marker)^18^. Cell-derived EVs were analyzed only in the total EV pool defined above. At baseline, the number of EryEVs was approximately fourfold lower in our patients compared with healthy subjects^12^, but approximately 20-fold higher than in other ICU patients^18^. However, EryEVs accounted for only 0.6% of total EVs in our study, compared with more than 10% reported in healthy subjects^12^. This observation is likely due to the combination of higher levels of total EVs and lower levels of EryEVs. Of note, in our study, GEVs accounted for 0.5%, myeloid EVs for 0.3%, MEVs for 0.04%, platelet-derived EVs for 0.4%, and activated platelet-derived EVs for 0.6% of total EVs.

Peters et al. showed that EryEVs peaked 2 h after transfusion in healthy subjects who had previously received a “hit” with LPS and decreased again 4 and 6 h after transfusion. In our patients, the increase in EryEVs began as early as 1 h after transfusion and peaked 2 to 3 h after initiation of PRBC transfusion. We focused on the time interval of the first 90 min after PRBC transfusion. Therefore, we cannot state whether or not total EVs and EryEVs returned to baseline values, and might have also missed a delayed effect of transfusion on plasma EV counts. Another study even reported a decrease in plasma EryEV concentration 2 days after transfusion in ICU patients with subsequent recovery to baseline^18^, which may reflect a biphasic, bidirectional course. In addition, PRBC transfusion in our study also increased plasma levels of total EVs and GEVs at all three time points after transfusion, as well as levels of activated PEVs at T2 (30 min after the end of transfusion).

A 30-fold dilution of EVs by transfusing one unit of PRBCs into the blood volume of an adult patient could theoretically be expected^12^. We observed that mean EV concentrations in plasma were equal (total EVs) or only fivefold decreased (EryEV) compared to concentration in PRBC units. The increase of EVs in plasma upon transfusion may, at least in part, be caused by indirect pathways or mechanical shear stress leading to increased shedding of EVs by circulating red blood cells and/or platelets. We report a positive correlation between the increase of total and EryEVs in plasma during PRBC transfusion and PRBC storage duration, as has been shown before in an in vitro study^32^. This implies that the aforementioned indirect mechanism may be more pronounced after prolonged storage of stored PRBC and warrant further investigation.

As in our previous study addressing eicosanoids dynamics in this setting, feasibility was an important focus of this subanalysis. We have already described the relatively high recruitment rate of approximately 10% in our study^20^, compared with 2% in another study examining PRBC transfusion in ICU patients^19^. Of note, immediate non-routine processing of samples was required, with two subsequent centrifugation steps and immediate freezing in liquid nitrogen, occupying at least one person for 4 h. The fact that sampling personnel were available around the clock clearly accelerated recruitment, as sampling was performed after hours and on weekends in nearly one third of the included patients. Because the timing of PRBC transfusion in the ICU is largely unpredictable, future larger studies examining EV dynamics in postoperative patients in the ICU would need to have trained staff available at all times. The prospective selection of patient groups (i.e., Aortic and LuTx) was particularly appropriate for LuTx because this group provided predictable case numbers. Although we did not study trauma patients this patient population may also be considered in future investigations assessing the effects of EVs on TRIM^33,34^.

Our study has several limitations. As this was a predefined subanalysis of a feasibility study, sample size is limited. In addition, we studied only a single-unit transfusion in ICU patients. Therefore, the data do not allow us to draw conclusions about the effect of transfusing multiple PRBCs. Transfusion of other blood products containing EVs (i.e., fresh frozen plasma and platelet concentrate) was not an exclusion criterion and may also have influenced our results. Transfusion of PRBCs within 12 h before was an exclusion criterion in our study, but transfusion of PRBCs outside of this time frame could have influenced our results. Furthermore, the main study focused on the recruitment of ICU patients and the detection of eicosanoids in plasma. Therefore, other parameters of interest in plasma such as hemolysis parameters (e.g., free hemoglobin), pro- and anti-inflammatory cytokines, and coagulation parameters were not investigated^35–37^.

This study did not focus on donor variation effects, which is an important issue in transfusion medicine^38,39^. Lastly, definition and guidelines for the analysis of EVs are highly debated and constantly under development^29^. Therefore, the comparison of our data with other studies in the field (e.g., studies applying other markers than LA and Calcein AM for total EVs) may be limited.

In conclusion, EVs were detectable in all PRBC and ICU patient plasma samples. Although we did not observe a storage-dependent increase of EVs in PRBCs, total EVs and some EV subtypes increased in patient plasma after transfusion. Future studies to investigate transfusion-related EV dynamics in ICU patients are warranted but may require examination of a larger number of patients.

### Supplementary Information

Supplementary Information.

Supplementary Table S3.

## Data Availability

All data analysed during this study are included in this published article and its Supplementary Information files. Raw data are available from the corresponding author on reasonable request.
