# Supplementary material for: Red blood cell transfusion-related dynamics of extracellular vesicles in intensive care patients: a prospective subanalysis
Source: Sci Rep. 2024 Jan 9;14:911. doi: 10.1038/s41598-023-48251-w (PMC10776840; doi:10.1038/s41598-023-48251-w)
Supplement: Supplementary file 1 — Supplementary Information. [file 41598_2023_48251_MOESM1_ESM.pdf]

Supplementary Material

Red blood cell transfusion-related dynamics of extracellular vesicles in intensive care patients – a prospective subanalysis

Pierre Raeven[1], Katharina Karlhofer[1,2], Larissa S. Sztulman[3], Jonas Brugger[4], Konrad Hoetzenecker[5], Christoph Domenig[6], Gerda Leitner[7], Martin Posch[4], David M. Baron[1†], Andreas Spittler[2†]*

[1]Department of Anesthesia, General Intensive Care, and Pain Management, Division of General Anesthesia and Intensive Care, Medical University of Vienna, Vienna, Austria
[2]Department of Surgery, Division of Visceral Surgery and Core Facility Flow Cytometry, Medical University of Vienna, Austria
[3]Department of Surgery, Division of Visceral Surgery, Medical University of Vienna, Vienna, Austria
[4]Center for Medical Statistics, Informatics, and Intelligent Systems, Section for Medical Statistics, Medical University of Vienna, Vienna, Austria
[5]Department of Thoracic Surgery, Medical University of Vienna, Vienna, Austria
[6]Department of Surgery, Division of Vascular Surgery, Medical University of Vienna, Vienna, Austria
[7] Department of Blood Group Serology and Transfusion Medicine, Medical University of Vienna, Vienna, Austria
[†] These authors contributed equally to this work and share senior/last authorship

*Corresponding Author: andreas.spittler@meduniwien.ac.at

Table S1. Panel arrangement of antibodies and fluochromes. CD, cluster of differentiation; EVs, extracellular vesicles; PS, Phosphatidylserine; PE, R-phycoerythrin; PC7, R-phycoerythrin Cy7.

Panel	Origin cell	Antibody	Antigen	Clone	Fluochrome	Manufacturer
1	Erythrocyte	Anti-CD235a	CD235a	11E4B- 7-6	PE	Beckman Coulter, Brea, CA, USA
	Myeloic cell	Anti-CD15	CD15	W6D3	PC7	Biolegend, San Diego, CA, USA
2	Granulocyte	Anti-CD66b	CD66b	REA306	PE	Milteny, Bergisch Gladbach, Germany
	Monocyte	Anti-CD14	CD14	HCD14	PC7	Biolegend, San Diego, CA, USA
3	Platelet	Anti-CD62p	CD62p	REA389	PE	Milteny, Bergisch Gladbach, Germany
	Platelet	Anti-CD41	CD41	P2	PC7	Beckman Coulter, Brea, CA, USA
	Total EVs	Lactadherine	PS		Alexa647	CellSystems GmbH, Troisdorf, Germany
	Total EVs	Calcein AM	PS - negative EVs		Calcein Green	Thermo Fisher Scientific, Waltham, MA, USA

Table S2. Extended patient characteristics. PRBC, packed red blood cell; OP, time point of operation; ICU, intensive care unit; T0, time point of PRBC transfusion relevant to this study; OR, operating room; FFP, fresh frozen plasma; PC, platelet concentrate; n.a., not applicable; LuTx, bilateral lung transplantation; v/a ECMO, veno-arterial extracorporeal membrane oxygenation. Table has been published previously[1] and was modified according to patients included in this subanalysis.

Group	Patient identification number	Hours since last PRBC	Extracorporeal treatment	OP to ICU admission (days)	ICU length of stay (days)	T0 to ICU discharge (days)	Salvaged blood (ml) given in OR	Total FFP (units) in OR	Total FFP (units) in ICU	Total PC (units) in OR	Total PC (units) in ICU	Blood type PRBC	Blood type patient
Aorta	6	n.a.	-	0	5	4	840	0	0	0	0	A-	A-
	34	20	-	0	3	2	0	0	0	0	0	A-	A-
	36	n.a.	-	0	3	1	800	0	0	0	0	0+	0+
	48	25	-	0	16	15	2153	0	0	0	0	A+	A+
	81	18	-	0	10	8	2800	0	0	0	0	A+	A+
LuTx	11	48	-	0	7	5	0	12	1	1	0	AB+	AB+
	12	20	v/a ECMO	0	19	18	1100	11	0	3	2	A+	A+
	14	81	-	0	10	7	239	8	0	8	0	A+	A+
	15	12	-	0	6	3	250	11	0	0	0	0+	0+
	25	58	-	0	24	21	0	19	0	1	0	0-	AB-
	45	264	-	-5	33	18	0	25	0	4	2	0+	0+ (M-)
	49	61	-	1	35	27	2600	47	0	1	0	A-	A-
	64	42	-	0	39	33	1580	21	0	1	0	A-	A-
	73	43	-	0	4	1	0	17	4	0	0	A-	A-
Comparison	17	80	-	0	21	16	0	7	2	0	0	0+	0+
	20	39	-	0	13	10	0	0	0	0	0	B+	B+
	43	58	Dialysis	-2	22	18	0	0	0	0	0	AB+	AB+
	46	75	-	-26	61	34	0	0	0	0	0	A+	A+
	61	24	-	0	53	9	794	19	0	3	0	0-	0-
	68	25	-	0	2	1	0	0	0	0	0	A+	A+
	79	13	-	0	5	4	0	0	0	0	0	A+	A+
	80	n.a.	-	0	1	0	0	0	0	0	0	0+	0+
	87	n.a.	-	0	8	6	0	0	0	0	0	A+	A+

Table S3. EV levels (event count /µl) in patient plasma at predefined time points (T-2, T-1, T0, T1, T2, T3) and in transfused PRBCs. Pat ID; patient identification number; LuTx, bilateral lung transplantation; PRBC, pack red blood cells; CD, cluster of differentiation; EV, extracellular vesicle.

See attached Table_S3.xlsx

Table S4. Correlation of change in plasma concentration of specific extracellular vesicle (EV) subtype populations between baseline and T1, T2, or T3, respectively, and their concentration in the transfused PRBC in the whole cohort. Table depicts rho correlation coefficient and 95% confidence intervals in brackets. *p<0.05.

EV Subgroup	Delta concentration		
	**T1 - baseline**	**T2 - baseline**	**T3 – baseline**
**Total EV**	-0.37 [-0.68; 0.05]	0.60 [0.25; 0.81]*	0.50 [0.11; 0.76]*
**CD235a^{+} EV**	0.55 [0.18; 0.78]*	0.40 [-0.02; 0.70]	0.08 [-0.34; 0.48]
**CD41^{+}CD62p^{-} EV**	0.36 [-0.06; 0.67]	0.28 [-0.15; 0.62]	0.12 [-0.31; 0.51]
**CD62p^{+} EV**	-0.05 [-0.45; 0.37]	-0.04 [-0.45; 0.38]	-0.04 [-0.45; 0.38]
**Cd66b^{+} EV**	0.36 [-0.06; 0.67]	-0.06 [-0.46; 0.36]	0.15 [-0.28; 0.53]
**CD14^{+} EV**	0.02 [-0.40; 0.43]	-0.13 [-0.52; 0.30]	-0.13 [-0.51; 0.30]

Figure S1. Scatter plot of EV counts analyzed in stored packed red blood cells (PRBCs, y-axes) plotted against the storage duration of the respective PRBCs (x-axes). Patients from Aorta group are labeled by triangles, patients from Lung transplantation group are labeled by squares, and patients from the Comparison group are labeled by circles. CD, cluster of differentiation; CI, confidence interval; EVs, extracellular vesicles; PRBCs, packed red blood cells; PLT, platelets.

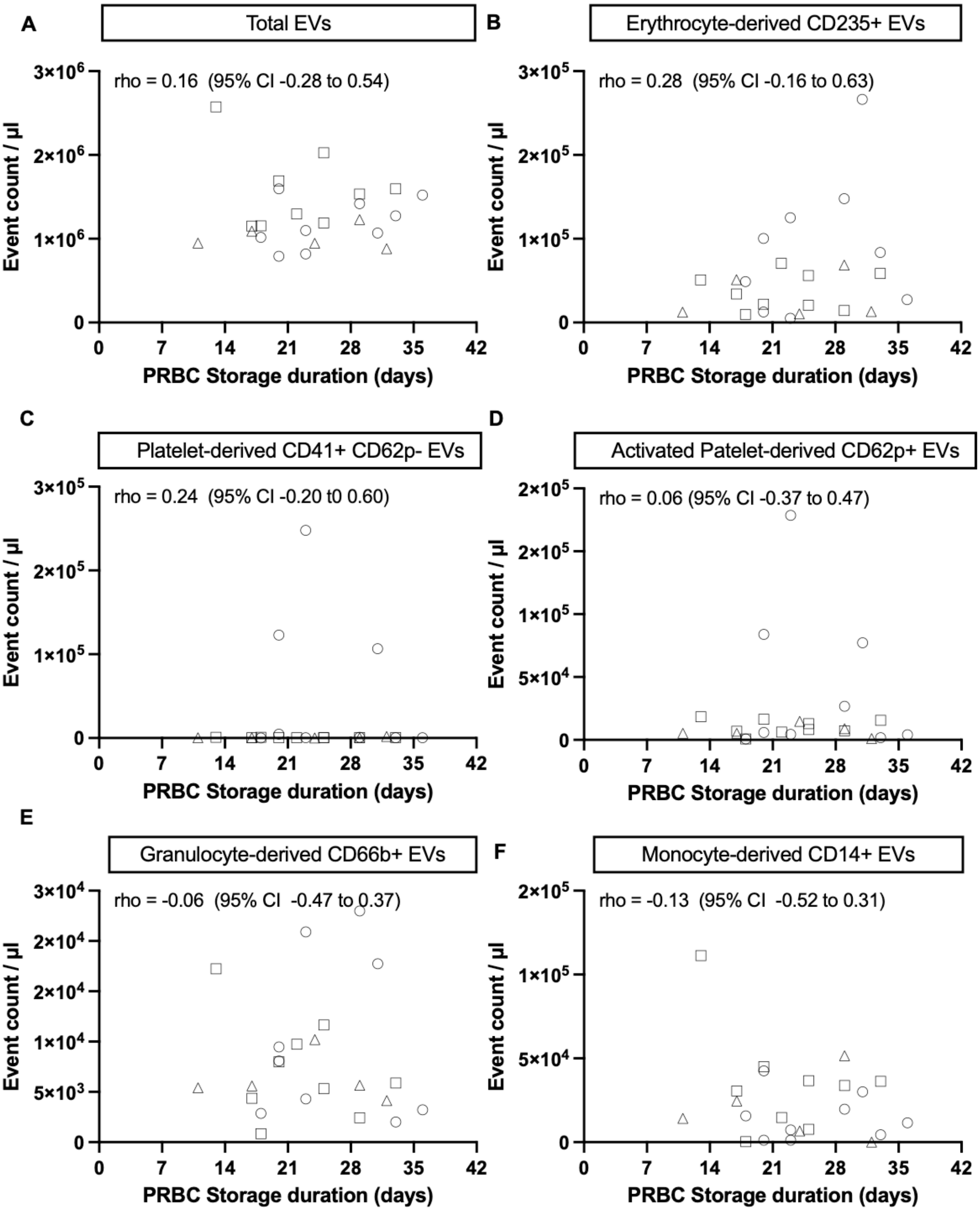

Figure S2. Individual EV time courses. Grey lines depict time courses of EV concentrations in plasma for individual patients separated according to study group. Red lines indicate the slopes of the EV concentrations in events per µl per hour after transfusion as estimated by linear regression, which are also depicted in the upper left corner of each panel. *p<0.05, **p<0.01 versus zero. LuTx, bilateral lung transplantation EV, extracellular vesicles; CD, cluster of differentiation.

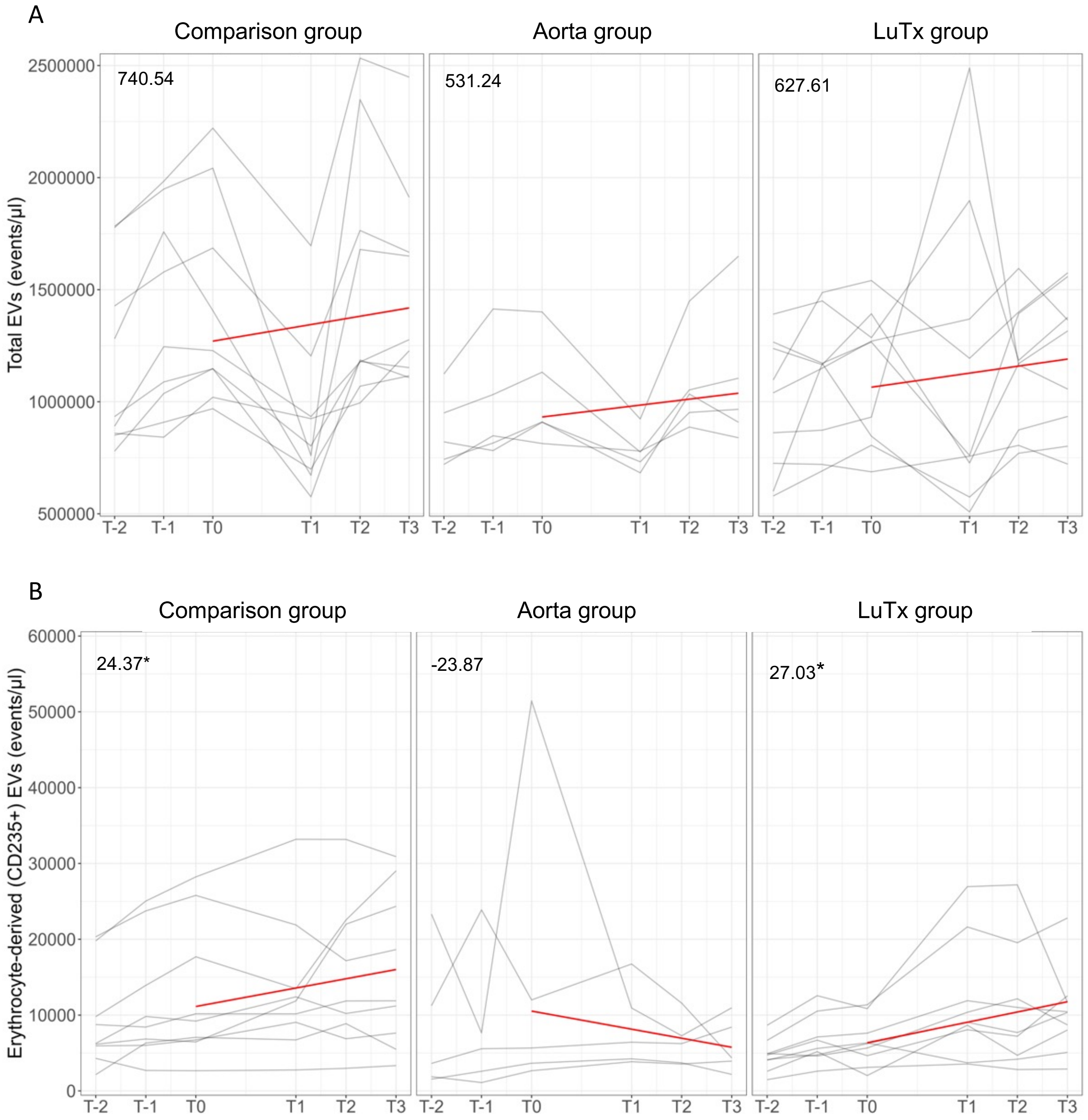

C

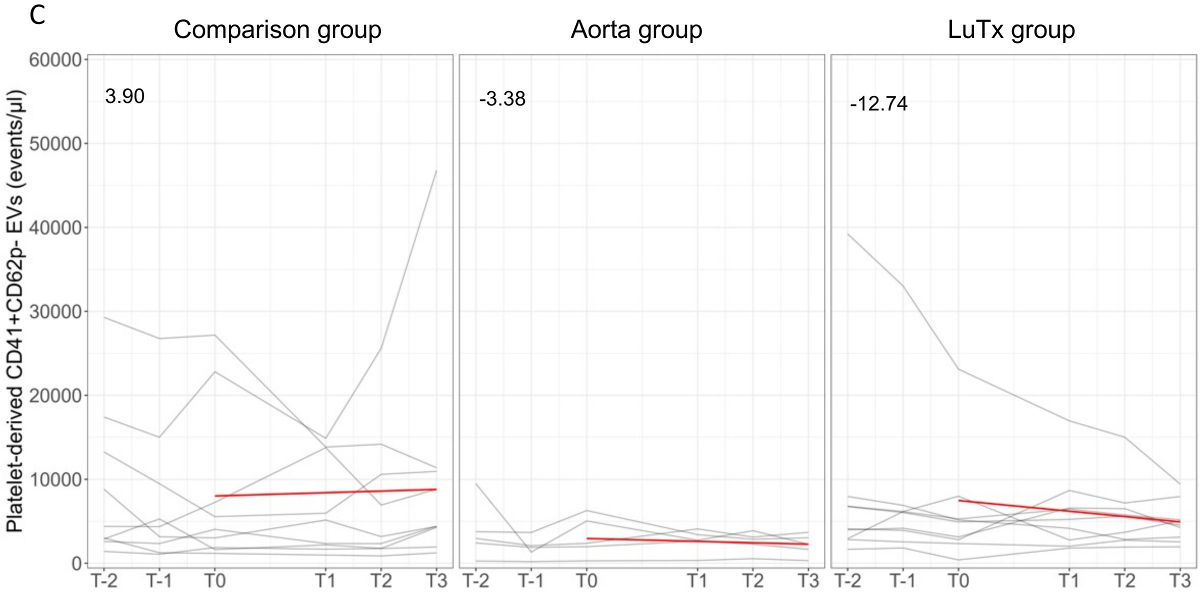
Comparison group
Aorta group
LuTx group
Platelet-derived CD41+CD62p- EVs (events/µl)
60000
50000
40000
30000
20000
10000
0
3.90
-3.38
-12.74
T-2 T-1 T0 T1 T2 T3

D

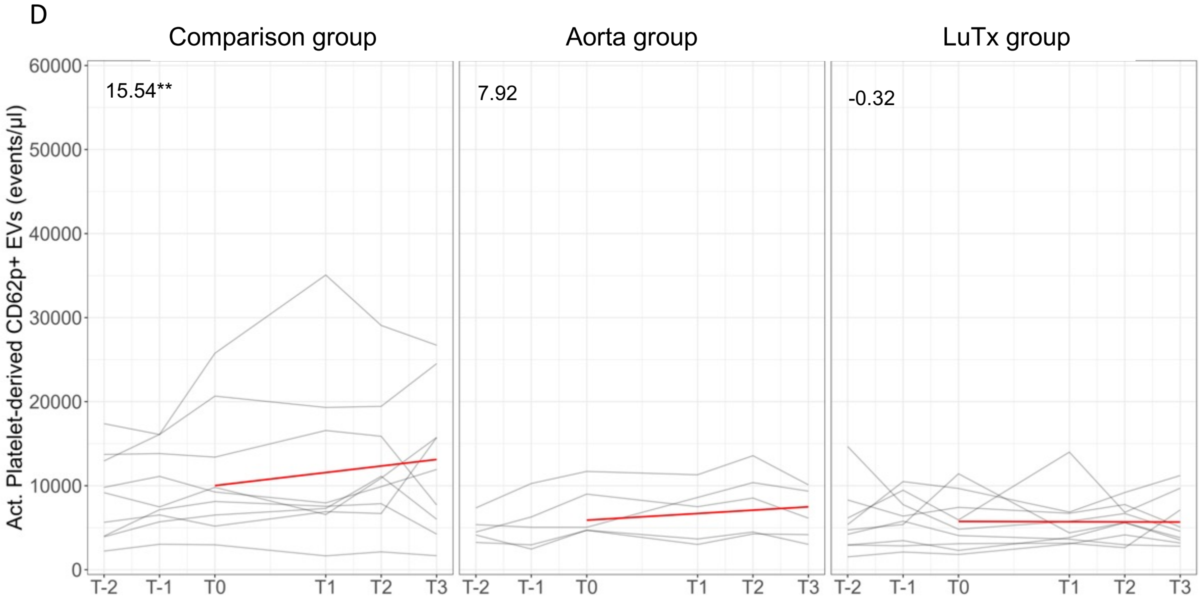
Comparison group
Aorta group
LuTx group
Act. Platelet-derived CD62p+ EVs (events/µl)
60000
50000
40000
30000
20000
10000
0
15.54**
7.92
-0.32
T-2 T-1 T0 T1 T2 T3

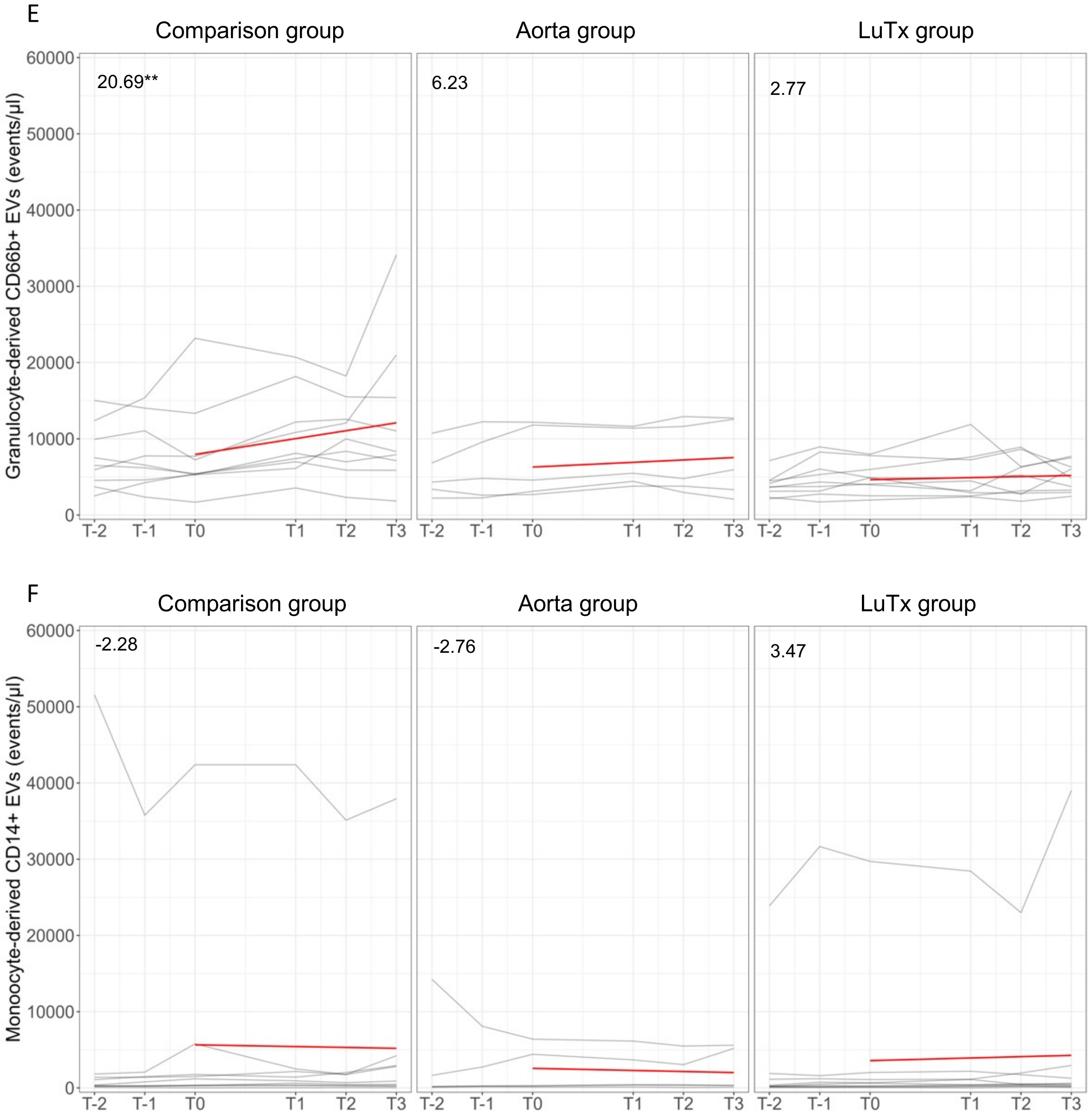
E
Comparison group
Aorta group
LuTx group
20.69**
6.23
2.77
Granulocyte-derived CD66b+ EVs (events/µl)
60000
50000
40000
30000
20000
10000
0
T-2
T-1
T0
T1
T2
T3
F
Comparison group
Aorta group
LuTx group
-2.28
-2.76
3.47
Monoocyte-derived CD14+ EVs (events/µl)

References

1. Raeven P, Hagn G, Niederstaetter L, et al. Red blood cell transfusion-related eicosanoid profiles in intensive care patients-A prospective, observational feasibility study. *Front Physiol.* 2023;14:1164926.
